# A Case of Angiomyolipoma Arising in the Tongue

**DOI:** 10.1155/2011/698139

**Published:** 2011-12-08

**Authors:** Shinya Yura, Shintaro Terahata, Shun Sugiguchi

**Affiliations:** ^1^Department of Oral and Maxillofacial Surgery, Tonami General Hospital, 1-61 Shintomi-cho, Tonami, Toyama 939-1395, Japan; ^2^Department of Clinical Pathology and Laboratory Medicine, Tonami General Hospital, 1-61 Shintomi-cho, Tonami, Toyama 939-1395, Japan

## Abstract

A 61-year-old woman was referred to our hospital with a mass in the left proglossis. The lesion was excised with a provisional diagnosis of hemangioma. The mass was well-demarcated and easily dissected with an ultrasonic surgical aspirator. The histopathologic diagnosis was angiomyolipoma arising in the tongue. There were no signs of recurrence at followup at 18 months.

## 1. Introduction

Angiomyolipoma is a benign tumor that is histologically composed of groups of mature adipose tissue intermixed with convoluted thick-walled blood vessels, interlacing bundles, and irregularly arranged sheets of smooth muscle, with the kidney as the most frequent site of involvement. Extrarenal angiomyolipoma can occur in organs such as the liver, lung, uterus, and skin. Oral and maxillofacial cases are rare. Only two instances of this tumor arising in the tongue have previously been reported [1, 2]. This paper describes a case of angiomyolipoma arising in the tongue.

## 2. Case Report

**Figure 1:**
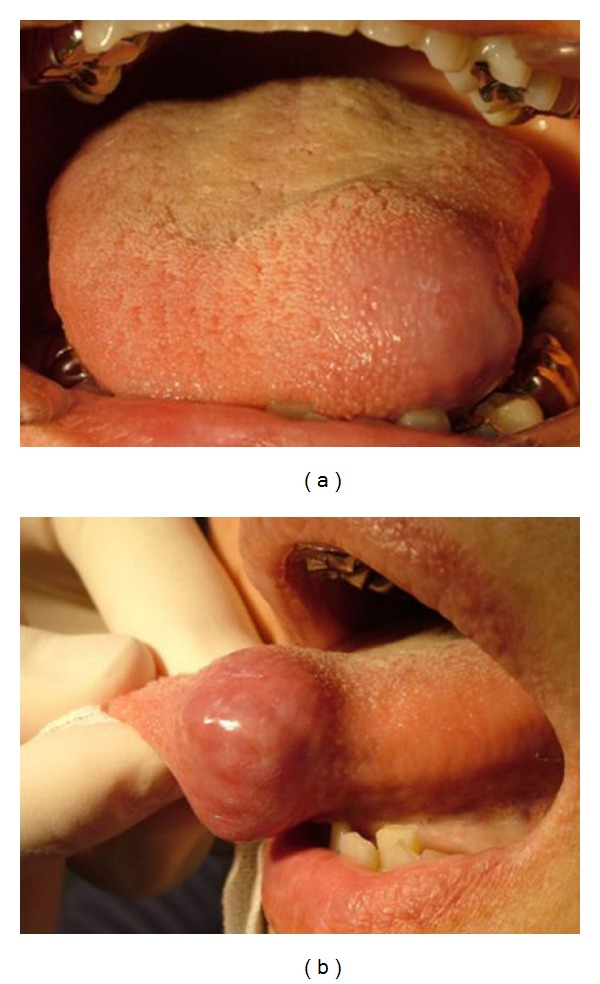
Clinical appearance of the lingual tumor. The mass is dome shaped with a dark violet-colored surface.

**Figure 2:**
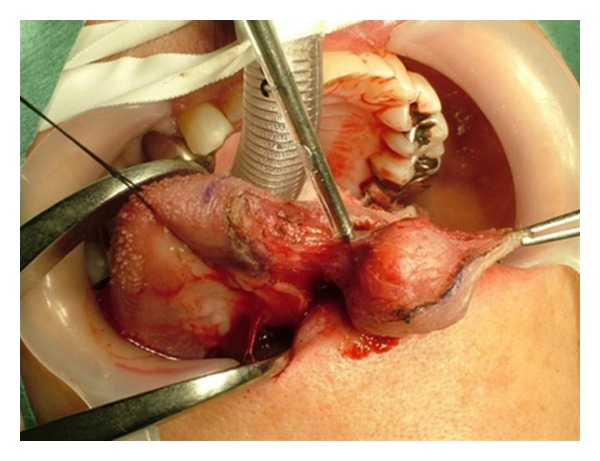
View of surgery. Resection of the tumor was performed using an ultrasonic surgical aspirator, which offers tissue dissection with easy intraoperative hemostasis.

**Figure 3:**
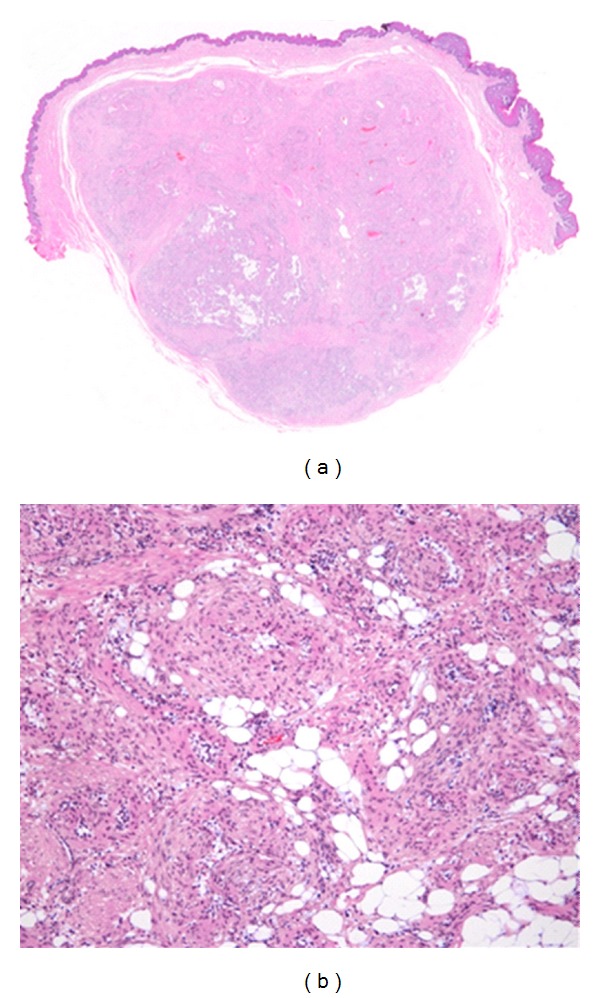
Photomicrograph of an angiomyolipoma of the tongue. (a) An encapsulated lesion composed of a proliferation of an intricate mixture of mature adipose tissue, blood vessels, and smooth muscle (H-E staining, magnification ×10). (b) Many large blood vessels with thick muscular walls (H-E staining, magnification ×100).

**Figure 4:**
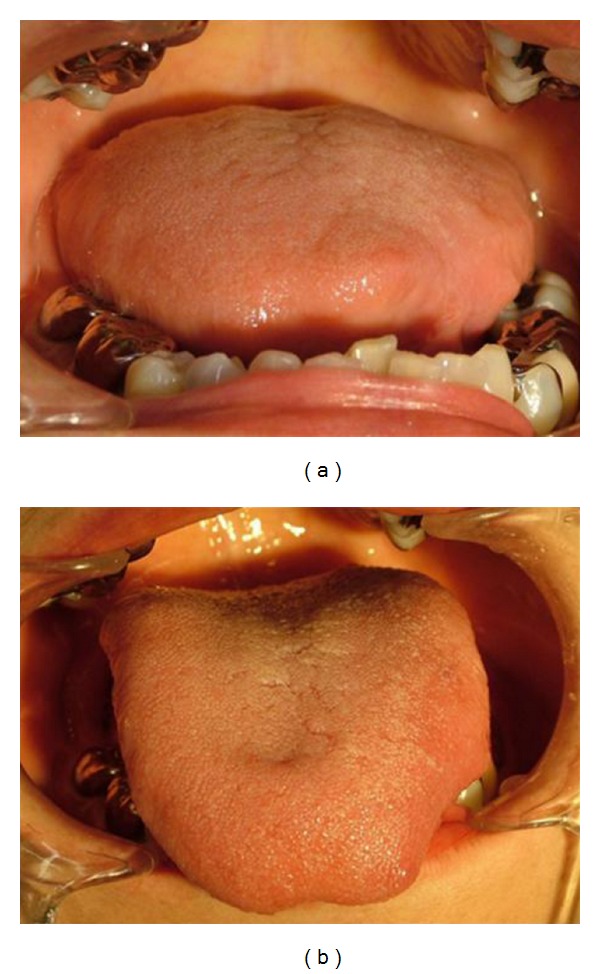
Postoperative intraoral view. The wound healed without any postoperative complications.

A 61-year-old woman was referred to our hospital with a mass in the left proglossis that was painless, but had enlarged slowly for 5 years. There were no clinical signs or family history of tuberous sclerosis. The mass measured 20 mm × 20 mm and was soft and dome shaped with a dark violet-colored surface (Figure 1). The lesion was excised with a provisional diagnosis of hemangioma. The mass was well-demarcated and easily dissected with an ultrasonic surgical aspirator (Figure 2). Histopathological examination showed an encapsulated lesion composed of a proliferation of an intricate mixture of mature adipose tissue, blood vessels, and smooth muscle (Figure 3(a)). The vascular component showed a proliferation of vessels of different types and sizes. Many large blood vessels with thick muscular walls were seen throughout the lesion. Mature adipose tissue was mixed thinly (Figure 3(b)). The histopathologic diagnosis was angiomyolipoma arising in the tongue. The patient's postoperative course was uneventful, and there were no signs of recurrence at followup at 18 months (Figure 4).

## 3. Discussion

Only two cases of angiomyolipoma arising in the tongue have previously been reported. Koizumi et al. [1] reported a case of angiomyolipoma in the centre of the tongue. Ide et al. [2] described a case of a angiomyolipomatous hamartoma arising in the left lateral border of the tongue. In the present case, angiomyolipoma arose in the proglossis and it had a diagnosis of hemangioma because of its hardness and color. Koizumi et al. [1] excised their mass with a provisional diagnosis of fibroma. Because the three principal components in angiomyolipoma, regardless of its location, vary greatly in proportion and distribution, its heterogeneity may cause diagnostic confusion. The differential diagnosis includes lipomatous or myolipomatous tumours, angiomyoma, angiolipoma, hemangioma, fibroma, and fibrolipomatous hyperplasia. A final diagnosis thus requires histopathological examination.

Angiomyolipoma could be considered a hamartoma, but there is no consensus that these lesions are a single entity. Ide et al. [2] reported that the present lesion may not be a classic oral angiomyolipoma because it was poorly circumscribed and not encapsulated. They suggested the term angiomyolipomatous hamartoma to designate this lesion. In agreement with Ide et al.[2], we diagnosed the present case as an angiomyolipoma because the mass had enlarged gradually with active proliferation and was well-encapsulated.

Oral angiomyolipomas are sharply demarcated and either completely or partially encapsulated. Previously, recurrence has not been documented. In our case, the mass was well-demarcated and easily dissected with an ultrasonic surgical aspirator. The patient has remained free of recurrence 18 months after excision. In particular, tongue cancer and hemangioma are tumors with many blood vessels in the proposed area of surgical excision. For preservation of the nervous system and avoidance of bleeding, an ultrasonic surgical aspirator is effective in the resection of these tongue tumors [3–5]. In our case, the wound healed without any postoperative complications such as scarring or bleeding.
